# Investigation of the protective role of *Ginkgo biloba* L. against phytotoxicity, genotoxicity and oxidative damage induced by Trifloxystrobin

**DOI:** 10.1038/s41598-024-70712-z

**Published:** 2024-08-27

**Authors:** Saliha Kesti, Oksal Macar, Tuğçe Kalefetoğlu Macar, Kültiğin Çavuşoğlu, Emine Yalçın

**Affiliations:** 1 Department of Biology, Faculty of Science and Art, Giresun University, Giresun, Turkey; 2 Şebinkarahisar School of Applied Sciences, Department of Food Technology, Giresun University, 28400 Giresun, Turkey

**Keywords:** *Allium cepa* L., Antioxidant, Genotoxicity, *Ginkgo biloba* L., Phenolic, Trifloxystrobin, Biochemistry, Biotechnology, Molecular biology, Plant sciences, Risk factors

## Abstract

Trifloxystrobin (TFS) is a widely used strobilurin class fungicide. *Ginkgo bilob*a L. has gained popularity due to its recognized medicinal and antioxidant properties. The aim of this study was to determine whether *Ginkgo biloba* L. extract (Gbex) has a protective role against TFS-induced phytotoxicity, genotoxicity and oxidative damage in *A. cepa*. Different groups were formed from *Allium cepa* L. bulbs subjected to tap water (control), 200 mg/L Gbex (Gbex1), 400 mg/L Gbex (Gbex2), 0.8 g/L TFS solution (TFS), 200 mg/L Gbex + 0.8 g/L TFS (TFS + Gbex1) and 400 mg/L Gbex + 0.8 g/L TFS (TFS + Gbex2), respectively. The phenolic composition of Gbex and alterations in the morphological, physiological, biochemical, genotoxicity and anatomical parameters were evaluated. Rutin, protocatechuic acid, catechin, gallic acid, taxifolin, p-coumaric acid, caffeic acid, epicatechin, syringic acid and quercetin were the most prevalent phenolic substances in Gbex. Rooting percentage, root elongation, weight gain, chlorophyll *a* and chlorophyll *b* decreased by approximately 50%, 85%, 77%, 55% and 70%, respectively, as a result of TFS treatment compared to the control. In the TFS group, the mitotic index fell by 28% compared to the control group, but chromosomal abnormalities, micronuclei frequency and tail DNA percentage increased. Fragment, vagrant chromosome, sticky chromosome, uneven chromatin distribution, bridge, vacuole-containing nucleus, reverse polarization and irregular mitosis were the chromosomal abnormalities observed in the TFS group. The levels of proline (2.17-fold) and malondialdehyde (2.71-fold), as well as the activities of catalase (2.75-fold) and superoxide dismutase (2.03-fold) were increased by TFS in comparison to the control. TFS-provoked meristematic disorders were damaged epidermis and cortex cells, flattened cell nucleus and thickened cortex cell wall. Gbex combined with TFS relieved all these TFS-induced stress signs in a dose-dependent manner. This investigation showed that Gbex can play protective role in *A. cepa* against the phytotoxicity, genotoxicity and oxidative damage caused by TFS. The results demonstrated that Gbex had this antioxidant and antigenotoxic potential owing to its high phenolic content.

## Introduction

Trifloxystrobin (TFS), [methyl(E)-α-methoxyimino-2-[(E)-1-(3-trifluoromethylphenyl) ethylidenaminooxymethyl] phenylacetate], is a fungicide of the strobilurin class that prevents mitochondrial respiration of fungi. Methoxyacrylate, the bactericidal active group of TFS, has a broad range, minimal toxicity and exceptional biological activity^1^. It attaches to the region (Qo) of complex III, halting the synthesis of adenosine triphosphate and upsetting the energy cycle of fungi^2^. The use of strobilurin fungicides is growing annually, and they currently have the highest global sales among fungicides^3^. TFS is extensively utilized for protecting grains, rice, fruits, soybeans, vegetables, lawns, stone fruits, grapes and potatoes. According to Saha et al.^4^, TFS residues in soil vary from 0.81 to 1.8 mg/kg. Fungicide residues that accumulate in the soil, air and water as a result of intense and continuous use of fungicides may have the potential to induce detrimental effects on the environment and human health^5^. Although strobilurin-class fungicides are thought to have low toxicity on non-target organisms, there are studies showing undesirable toxic and genotoxic effects of TFS^3,6^.

*Ginkgo biloba* L. is the best-known "living fossil" in the world from the Mesozoic era. It is the only species that today natively represents the members of the *Ginkgoaceae* family^7^. Although the ginkgo tree is native to China, Japan and Korea, it is currently found in many locations in America, Argentina, India, Europe and New Zealand^8^. *G. biloba* has gained popularity all around the world due to its extraordinary adaptability to the environment as well as its important ornamental and medicinal virtues^9^. The plant is so resistant to biotic and abiotic stresses that it was the first plant to germinate after the atomic bomb explosion in Hiroshima^10^. As a noteworthy representative of culinary and traditional plants, *G. biloba* provides extraordinary benefits to modern medicine with its antioxidant, antihypertensive, antidiabetic, antilipidemic, hepatoprotective, memory-enhancer and cardioprotective effects^9,11^. The therapeutic capacity of *G. biloba* leaf is derived from its carboxylic acids, flavonoids, catechins, procyanidins, terpenoid lactones, lignans, alkyl phenolic acids, polysaccharides and other phytochemicals. Furthermore, the *G. biloba*-derived extracts comprise several pharmaceutically important ginkgolides, ginkgolic acid, bilobalide, flavonoids and glycosides^11^. Flavonoids are vital antioxidants among several polyphenols and they fight against intracellular oxidative stress-related diseases by chelating heavy metals thanks to their phenolic nature^12^.

The use of plant-based assays has become a valuable, cost-effective and accessible approach for the identification of cytotoxicity, mutagenicity, genotoxicity and clastogenicity^13^. Several environmental organizations, including the World Health Organization and the United Nations Environmental Program, encourage the utilization of plants to assess the toxicity of pollutants^14^. The *Allium cepa* root test is deemed to be the most successful assay among cyto-genotoxicity monitoring systems. *A. cepa* L. is a very effective model for biomonitoring genotoxicity due to its huge and discernible chromosomes (2n = 16). Furthermore, an excellent link between plant and mammalian cell systems is provided by the physical similarity of *A. cepa* chromosomes to those of mammalian cells^15^. Another advantage of the *Allium* test is that the bulbs can produce a large number of adventitious roots from the disc stem in a very short time. Furthermore, the abundance of well-stainable cells and its suitability for the comet test are other important advantages of *A. cepa*^16^. Root growth retardation and a lower mitotic index (MI) in the *A. cepa* test are reflectors of toxicity and genotoxicity, while chromosome abnormalities (CAs) and micronuclei (MN) are indicators of genotoxicity and mutagenicity, respectively^17^.

The objective of this study was to ascertain whether Gbex exerts a protective effect against TFS-induced phytotoxicity, genotoxicity and oxidative damage in the *A. cepa* model plant. Therefore, TFS and Gbex-induced changes in morphological and physiological (rooting percentage, root elongation, weight gain), cytogenetic (CAs intensity, MN formation, MI alteration, DNA tail occurrence) and biochemical (malondialdehyde level, superoxide dismutase activity, catalase activity, the concentrations of chlorophyll *a*, chlorophyll *b* and proline) parameters in *A. cepa* bulbs were examined. Cross-sections of the roots were employed to screen for meristematic cell disorders. The evaluation of phenolic compounds in the composition of Gbex was also included in the study.

## Materials and methods

### Preparation of test materials and solutions

Freshly harvested, pesticide-free *A. cepa* bulbs with an average weight of 11 ± 1.5 g were acquired commercially. Healthy and similarly shaped bulbs were directly exposed to the relevant media after being stripped of their outer scales. Trailer, a commercial product made by Hektaş Group (Kocaeli–Türkiye) that contains 50% TFS, was diluted with water to prepare an aqueous TFS solution. The other chemicals employed in the present research were of analytical grade. Additive-free Gbex containing 100% pure *G. biloba* leaf used in the study was purchased commercially from SepeNatural. The extract has the following certificates:

- T.C. Ministry of Food, Agriculture and Livestock Sepe Natural Business Registration Number TR-35-K-000853
- FDA Food and Drug Administration Sepe Natural Business Registration Number 12568327256
- GMP (Good Manufacturing Practice)
- ISO 22000:2005 (HACCP Hazard Analysis and Critical Control Points standards and Food Safety Management System

Leaf samples were extracted with ethanol. Ethanol is a solvent that has been widely and successfully used in preparing similar herbal extracts. Although the solvent was completely evaporated at the final stage of extract preparation, ethanol was preferred because it is the safest option for living organisms compared to other solvents.

**Fig. 1:**
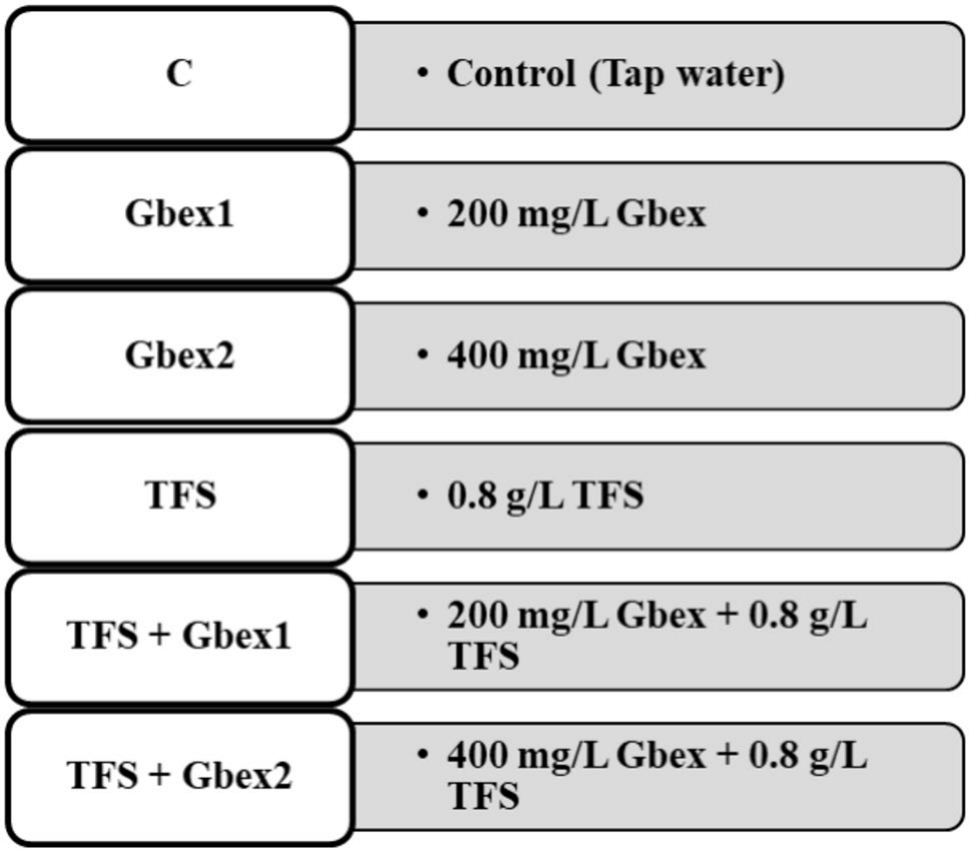
Experimental groups and test concentrations.

The TFS dose used in this study was determined according to the study of Macar et al.^6^, in which TFS doses were determined by considering rooting rate and root elongation. Experimental solutions were prepared using water. The doses of Gbex were established in accordance with the recommended daily dose and the literature reporting doses at which strong protection and beneficial effects were observed^18–20^. In this context, two doses of Gbex were used in the study: 200 mg/L and 400 mg/L. Six groups with fifty onions each were established in order to investigate the effects of TFS and Gbex (Fig. 1). One of these groups was designated as the control group and treated with tap water. The other groups (Gbex1, Gbex2, TFS, TFS + Gbex1 and TFS + Gbex2) were exposed to 200 mg/L Gbex, 400 mg/L Gbex, 0.8 g/L TFS, 200 mg/L Gbex + 0.8 g/L TFS and 400 mg/L Gbex + 0.8 g/L TFS, respectively. The whole experimental process continued in the dark until the completion of three cell cycles (3 days) in onion roots. Subsequently, the roots were harvested and all analyses except chlorophyll analysis were performed. Samples for the determination of chlorophyll were collected at the conclusion of the sixth day. Solutions were prepared by dissolving the appropriate amounts of extract and/or TFS in tap water. The solutions in contact with the bulbs were freshly prepared and refreshed daily to avoid concentration changes. Experimental research on plants and plant parts (onion bulbs) complies with relevant institutional, national and international guidelines and legislations.

### Phenolic profile of Gbex

*Ginkgo biloba* samples (1 g) were extracted with a 4:1 methanol-dichloromethane solvent (4:1). The extract was filtered with a 0.45 µm sterile syringe and the filtrate was used in phenolic substance analysis. The ODS Hypersil 4.6*250 mm column was used in the analysis performed on the LC–MS/MS (Thermo Scientific) device. The elution gradient consisted of mobile phase A (water with 0.1% formic acid) and mobile phase B (methanol). The gradient program was set as follows 0–1 min, 0% B; 1–22 min, 95% B; 22–25 min, 95% B; 25–30 min, 100% B. The total evaluation time was 34 min. The injection volume and the solvent flow rate were set at 20 µL and 0.7 mL/min, respectively^21,22^. LC–MS/MS analysis was performed at HUBTUAM-Hitit University.

### Assessment of morphological and physiological traits

Root length and weight gain analyses were performed based on the values of 10 bulbs, while 50 bulbs were evaluated to determine the rooting percentage (RP). Bulbs having adventitious roots that grew more than 10 mm were included in the calculation of RP (Eq. 1).

1 $$ {\text{RP}}(\% ) = \left( {{\text{number of rooted bulbs}}/{\text{number of total bulbs}}} \right) \times 100 $$

The length of the roots was measured from the base to the tip with the help of a digital caliper.

The amount of weight increase was quantified by calculating the difference between the bulb weights at the beginning and end of the experiment. All bulbs included in the analyses of root length and weight gain were chosen at random. The root length and weight gain analyses were performed in ten replicates.

### Assessment of genotoxicity and cytotoxicity

Analyses of CA intensity, MN formation, MI alteration and DNA tail occurrence were carried out to track the changes in cytotoxicity and genotoxicity parameters. Some of the adventitious roots from the bulbs that were around the same length were used for the cytotoxicity and genotoxicity assays. The first three analyses were performed on the same slides prepared according to the procedure of Macar et al.^6^, while the Comet test methodology of Chakraborty et al.^23^ was used to monitor the last one. Following the experiment, 1-cm sections of the root ends were cut off and immersed in Clarke liquid (3/1: ethanol/glacial acetic acid) for 120 min. Following fixation, washing was carried out with a 96% ethyl alcohol solution for 15 min. Root pieces were hydrolyzed in 1 N HCl at 60 °C for 9 min. Root tissues were then transferred into 45% glacial acetic acid. Roots were kept in this acidic medium for 30 min. They were then rinsed with distilled water to remove residues. The root material was squashed between the slide and the coverslip and transformed into a preparation material following a 12-h staining procedure with an acetocarmine (1%) solution. After this point, a microscope (Irmeco, IM-450 TI) was employed to screen the CAs (from 100 cells on 10 slides, 1000 cells overall), MN (from 100 cells on 10 slides, 1000 cells overall) and MI (from 1000 cells on 10 slides, 10,000 cells overall) scores. For the acceptance of objects seen outside the nucleus as MN, the criteria of Fenech et al.^24^ were considered. According to these criteria, the diameter of the MN should be approximately one third of the nucleus diameter. The MN should be almost the same color as the main nucleus. The MN should not look like a bud of the nucleus; the MN and nucleus should be separated by a clear border. CA intensity, MN formation and MI alteration analyses were performed in ten replicates.

The MI is calculated using the following formula^25^;

2 $$ {\text{MI}} = \left[ {({\text{Number of cells in mitosis}})/({\text{Total number of cells scored}})} \right] \times 100 $$

DNA tail occurrence was evaluated through the comet assay. DNA was isolated using the instructions of Sharma et al.^26^. According to the procedure described by Dikilitaş and Koçyiğit^27^, slides that would be monitored in the comet test were prepared by soaking them in ethanol for a day before being dried in an oven. A coverslip was placed on top of the slide after it had been coated with the normal melting point agarose at a concentration of 1%. Following the complete solidification of the first layer, a 1:7 ratio of 1% low melting point agarose and freshly isolated cell solution are combined to create a second layer. A coverslip was set on the slide following the application of the second layer. The slides were placed in the freezer for 5 min before being placed in the electrophoresis tank for 40 min to allow the DNA to breakdown and release. To balance the alkaline environment after electrophoresis, the slides are three-rinsed in Tris–HCl (0.4 M, pH 7.5) for 5 min at 25 °C. Fluorescence microscopy was utilized to analyze the slides that were stained with 80–100 µl of ethidium bromide. The analysis of the comet was carried out using “TriTek 2.0.0.38 Automatic Comet Assay Software”. DNA fragments were divided into head and tail sections and the proportion of DNA (%) in each section was calculated. Based on the scale developed by Pereira et al.^28^, the percentage of tail DNA was used to assess the degree of DNA damage in the samples. The Comet scale gauges DNA damage using the tail DNA proportion as a reference: ≤ 5%: negligible; 5–20%: weak damage; 20–40%: medium damage; 40–75%: substantial damage; ≥ %75: massive damage. DNA tail analyses were conducted in ten replicates.

### Assessment of biochemical parameters

The use of bulbs with recently emerging green leaves allowed for the extraction of chlorophyll *a* (Chl *a*) and chlorophyll *b* (Chl *b*)^29^. A cold leaf sample weighing 0.1 g was placed into 2.5 mL of 80% acetone during the technique, which was maintained in complete darkness. This mixture was filtered, and the filtrate was then combined with 2.5 mL of 80% acetone. The supernatant was obtained after centrifugation at 3000 rpm in order to estimate the chlorophyll content read at 645 and 663 nm by a UV/VIS spectrophotometer. Chl *a* and Chl *b* amounts were computed considering the equation offered by Witham et al.^30^.

The technique of Bates et al.^31^ was employed to evaluate the free proline accumulation of the roots. A root segment weighing 0.25 g was homogenized in 5 ml of 3% sulfosalicylic acid. Following a filtration process achieved through Whatman No. 2 filter paper, the same volumes (2 ml) of filtrate, acid-ninhydrin and glacial acetic acid were placed in a hot water bath at 100 °C for 60 min. The mixture was submerged in icy water to halt the reaction. A 4 ml of toluene was added to the cooled mixture before it was shaken well for 5–20 s. This led to the aspiration of the toluene-chromophore fraction from the aqueous phase. The absorbance of the chromophore was then determined at 520 nm at the temperature of the room. Toluene was used as a blind. Using a standard curve and Eq. 3, proline contents were determined as fresh weight.

3 $$ \begin{aligned} & [\left( {\upmu {\text{g proline}}/{\text{ml}} \times {\text{ml toluene}}} \right)/115.5\;\upmu {\text{g}}/\upmu {\text{mole}}]/[({\text{g sample}})/5] \\ & \quad =\upmu {\text{moles proline}}/{\text{g of fresh weight root material}}. \\ \end{aligned} $$

MDA, the most prevalent aldehyde of oxidative stress-related lipid oxidation in membranes, was determined spectrophotometrically^32^. To separate the supernatant, a root segment weighing 1 g was centrifuged in 2 ml of 5% trichloroacetic acid. At 12,000 rpm and ambient temperature, the procedure took 15 min. For 28 min, the same volumes of supernatant and 20% TCA + 0.5% thiobarbituric mixture were allowed to boil at 96 °C. The reaction between the supernatant and the mixture was halted by transferring the tubes to an ice-cold water tank. At 10,000 rpm and ambient temperature, a further centrifugation process (5 min) was performed. Calculating the concentration of MDA (µM/g FW) in the samples was then achieved by reading the supernatant's absorbance at 532 nm.

Antioxidant enzyme activities (SOD and CAT) were evaluated in addition to MDA accumulation to comprehend the impact of oxidative stress on the root cells of *A. cepa*. Two enzymes were extracted using precisely the same method^33^.

In order to measure the SOD enzyme activity, a reaction solution containing 1.5 mL of 0.05 M sodium phosphate buffer (pH 7.8), 0.3 mL of 130 mM methionine, 0.3 mL of 750 µM nitroblue tetrazolium chloride, 0.3 mL of 0.1 mM EDTA-Na_2_, 0.3 mL of 20 µM riboflavin, 0.01 mL of supernatant, 0.01 mL of 4% polyvinylpyrrolidone and 0.28 mL of deionized water was prepared. The reaction solution was then placed in front of two 15-W fluorescent lamps for 10 min. The tubes were transferred to a dark chamber for 10 min to terminate the reaction^34^. The level of SOD activity was quantified as units per milligram of fresh weight (U/mg FW) by measuring the absorbance at a wavelength of 560 nm^33^.

The CAT activity was measured using a total volume of 2.8 mL reaction mixture, which consisted of 0.3 mL 0.1 M H_2_O_2_, 1.5 mL 200 mM sodium phosphate buffer and 1.0 mL deionized water. The reaction required to measure CAT enzyme activity by monitoring the decrease in absorbance at a wavelength of 240 nm, resulting from the consumption of hydrogen peroxide, was initiated by the addition of 0.2 mL of the enzyme extract to the mixture^35^. The final unit for CAT enzyme activity was calculated as OD240 nm min/g FW. All biochemical analyses were performed in ten replicates.

### Assessment of meristematic cell disorders

Chemical residues were removed by rinsing the roots with distilled water. A cross-section of *A. cepa* roots was taken from randomly chosen bulbs to determine the meristematic cell disorders induced by TFS. The samples were then stained with a drop of 5% methylene blue and the images captured by the IM-450 TI light microscope were used in the investigations. For each group, 100 images were examined and anatomical abnormalities were scored by two different observers. The damage frequency for each of the 100 images was expressed as follows: (−): 0–10 damage, (+): 10–25 damage, (++): 25–50 damage, (+++): 50–75 damage, (++++): 75–100 damage.

### Statistical assessment

The data from the study were statistically analyzed using SPSS Statistics V 23.0 software (IBM Corp., USA, 2015). The data are presented as the mean with a standard deviation (SD). The statistical significance between means was assessed using one-way ANOVA and Duncan tests. It was deemed statistically significant at *p* < 0.05.

## Results and discussion

**Fig. 2:**
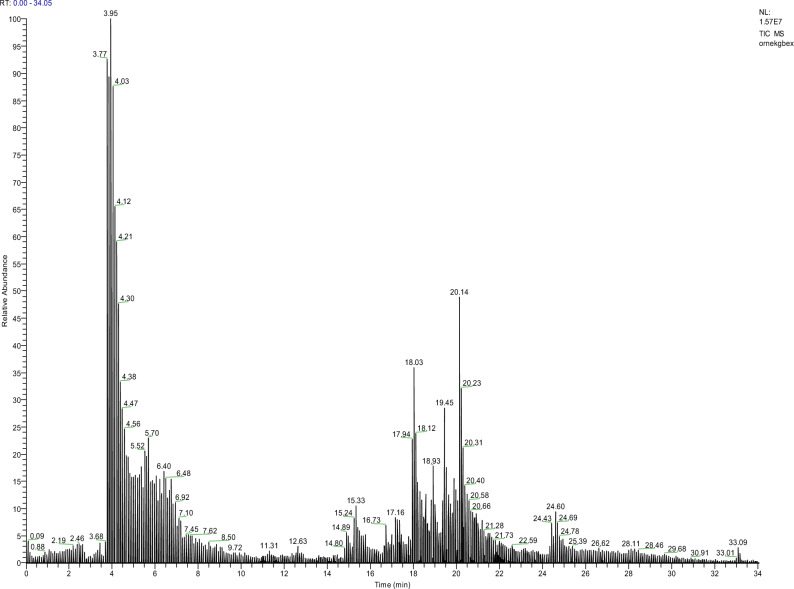
LC–MS/MS chromatogram of Gbex.

The bioactive chemicals present in Gbex were first identified in order to interpret the protective potential of Gbex against TFS (Fig. 2, Table 1). The most abundant phenolic compounds in the extract were rutin (41.54%), protocatechuic acid (38.24%), catechin (5.97%), gallic acid (5.20%), taxifolin (4.88%), p-coumaric acid (1.53%), caffeic acid (1.05%), epicatechin (0.58%), syringic acid (0.54%) and quercetin (0.42%). The phenolic compounds detected in the Gbex content have various biological and pharmacological properties, and these properties provide high protective effects. Rutin, one of the major compounds in the Gbex, contains a high − OH group and exhibits antioxidant activity by directly scavenging free radicals^36^. Protocatechuic acid, which is 38.24% in the Gbex, exhibits antioxidant activity by scavenging free radicals, preventing free radical formation and chelating transition metal ions^37^. Gallic acid, another phenolic found in the extract, is a strong antioxidant with a triphenolic structure^38^. All these active phenolic compounds detected in the content enable Gbex to exhibit various biological activities and to play a protective role. In the literature, Tabassum et al.^10^ described the functional phytochemicals present in *G. biloba* and reported that these substances are the basis for the numerous therapeutic and pharmacological activities of its extract. Liu et al.^39^ stated that the major active components of *G. biloba* leaves were flavonoids, phenolic acids and terpene lactones. Indeed, *G. biloba* contains 110 distinct flavonoids and flavonoid derivatives. According to Li et al.^11^, gallic acid, epigallocatechin, catechin and epicatechin are four different forms of flavonoids known as catechins that can be isolated from *G. biloba.*

**Table 1:** Phenolic compounds in Gbex content and their presence rates.

Compound	mg phenolic/kg	Presence rates of compounds (%)
Rutin	360.67	
Protocatechuic acid	332.006	
Catechin	51.861	
Gallic acid	45.212	
Taxifolin	42.37	
p-coumaric acid	13.31	
Caffeic acid	9.15	
Epicatechin	5.118	
Syringic acid	4.74	
Quercetin	3.67	
Vannilin	ND	
Ferulic acid	ND	
4-OH Benzoic acid	ND	
Salicylic acid	ND	
Rosmarinic acid	ND	
Oleuropein	ND	
Kaempferol	ND	
Resveratrol	ND	
Ellagic acid	ND	
Flavone	ND	
Protocatechuic aldehyde	ND	
Sesamol	ND	

The morphological and physiological alterations observed in *A. cepa* root cells are shown in Table 2. When compared to the control group, the 200 mg/L and 400 mg/L Gbex treatments did not result in any statistically significant differences (*p* > 0.05) in the rooting percentage, root length, or weight gain. Therefore, the Gbex dosages used in the study were not physiologically toxic. Çavuşoğlu et al.^40^ also reported that Gbex had no adverse impacts on the germination and growth of *Vicia faba* seeds. However, the emergence of new roots in bulbs was reduced by up to 50% after 0.8 g/L TFS administration. Similarly, TFS treatment caused a 85% and 77% reduction in root elongation and weight gain, respectively. Our results supported the findings of the earlier investigation by Macar et al.^6^, which showed the growth-inhibitory impacts of TFS treatment on *A. cepa* bulbs. TFS interferes with the transfer of electrons from cytochrome b to cytochrome c1 by attaching to the Qo site of cytochrome b^41^. The energy required for growth is not provided as a result of this restriction of mitochondrial oxidative respiration. In addition, Huang et al.^42^ reported that this peculiar action mechanism of TFS impedes growth by inhibiting oxidative phosphorylation in non-target organisms. It has also been reported that TFS can cause inhibition of the expression of growth-related genes in non-target organisms^43^. In addition, the processes that control cell wall elongation, loosening during the differentiation stage, and cell proliferation are prerequisites for the rooting process^44^. Disturbances in these processes or difficulties in the transfer of water and minerals required for these stages may also be associated with TFS-induced growth reduction. In comparison to the group that received TFS alone, Gbex treatment in a mixture with 0.8 mg/L TFS boosted rooting, root elongation and weight gain. As the dose of Gbex in the TFS and Gbex mixture rose, more restoration in growth was observed, but the results never matched those of the control group. Indeed, weight gain and root elongation in the TFS + Gbex2 group were 2.6 and 3.2 times those in the TFS group, respectively. Previous studies have shown that Gbex has selective effects on the activity of mitochondrial enzymes that construct the electron transport system, regulating mitochondria^45^. Very few studies in the literature show that *G. biloba* extract alleviates the growth suppressive-effects of stress factors in *A. cepa* ^46^. According to Çavuşoğlu et al.^40^, oxidative stress may be the major cause of xenobiotic-induced growth suppression in plants, and the antioxidant content of Gbex helped to alleviate this retardation.

**Table 2:** Protective efficiency of Gbex against TFS-induced morphological and physiological toxicity.

Groups	Rooting percentage (%)	Root length (cm)	Weight gain (g)	Before weight (g)	After weight (g)
(C) Control	100	9.8 ± 1.75^a^	+ 7.54^a^	11.52 ± 1.16	19.06 ± 1.85
Gbex1	100	9.6 ± 1.72^a^	+ 7.68^a^	11.74 ± 1.20	19.42 ± 1.88
Gbex2	100	10.4 ± 1.78^a^	+ 7.72^a^	11.36 ± 1.12	19.08 ± 1.82
TFS	50	1.5 ± 0.58^d^	+ 1.75^d^	11.66 ± 1.16	13.41 ± 1.28
TFS + Gbex1	63	2.9 ± 0.92^c^	+ 2.90^c^	11.83 ± 1.25	14.73 ± 1.35
TFS + Gbex2	75	4.8 ± 1.35^b^	+ 4.53^b^	11.59 ± 1.18	16.12 ± 1.46

C: Control, Gbex1: 200 mg/L Gbex, Gbex2: 400 mg/L Gbex, TFS: 0.8 g/L TFS, TFS + Gbex1: 200 mg/L Gbex + 0.8 g/L TFS, TFS + Gbex2: 400 mg/L Gbex + 0.8 g/L TFS. Values (displayed as mean ± SD) in the same column with a different letter (a–d) are significant at *p* < 0.05. Germination percentage was calculated considering 50 bulbs and 10 bulbs were utilized to determine root length and weight gain.

**Fig. 3:**
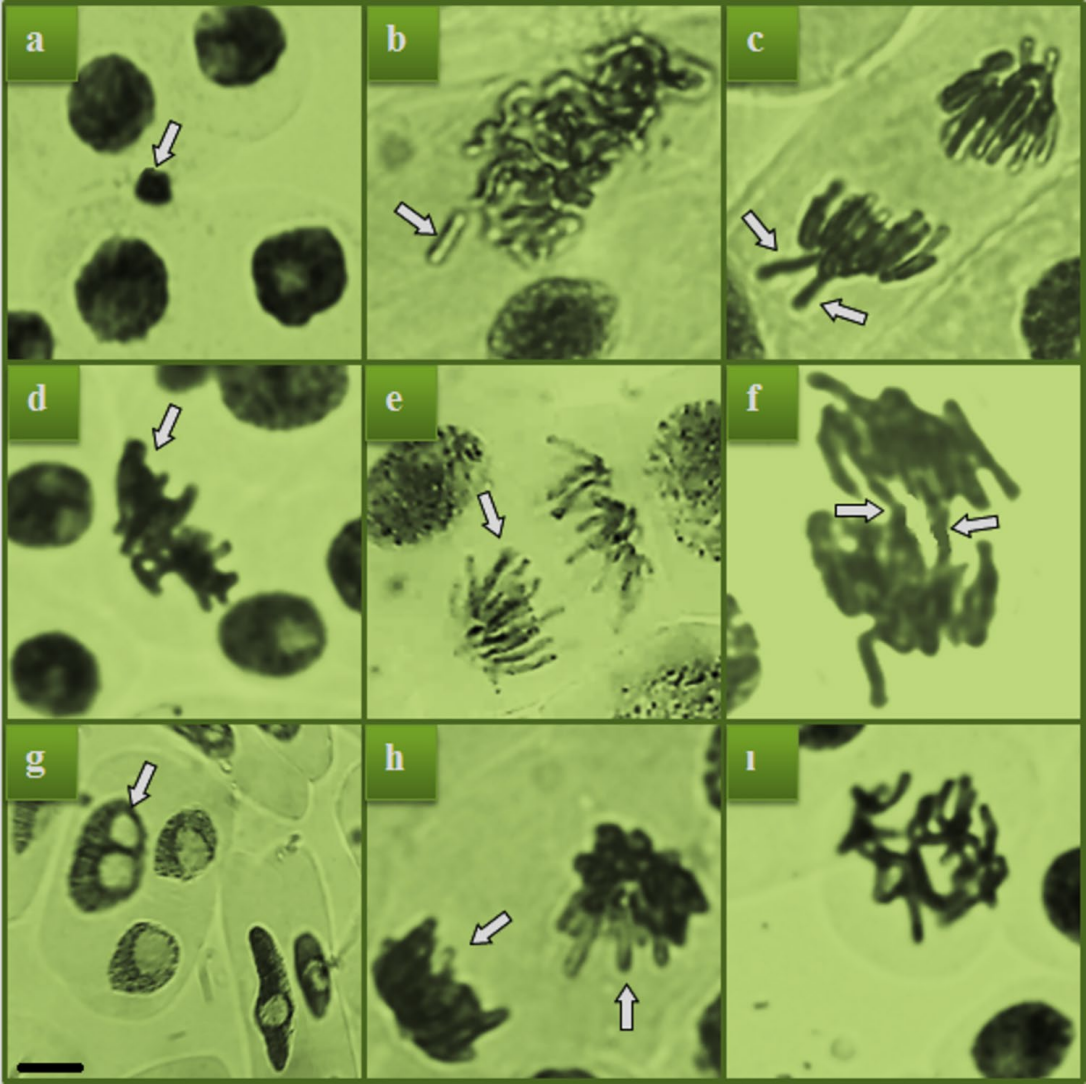
Chromosomal aberrations identified following TFS treatment. MN (**a**), fragment (F) (**b**), vagrant chromosome (V) (**c**), sticky chromosome (S) (**d**), uneven chromatin distribution (UCD) (**e**), bridge (B) (**f**), vacuole-containing nucleus (VCN) (**g**), reverse polarization (RP) (**h**), irregular mitosis (IM) (**i**). Scale bar: 10 µm.

CAs, which were extremely rare in the control group, might arise from spontaneous DNA changes (Table 3). Gbex treatments had no statistically meaningful impact on MI, MN, or CA amounts (*p* > 0.05) in comparison to the control group. Therefore, the extract doses employed were not genotoxic. TFS administration, on the other hand, brought about a notable drop in MI and a considerable rise in MN and CAs (*p* < 0.05) (Fig. 3, Table 3). MI in the TFS group was 28% lower than in the control group. MI is used to gauge cell division as well as material toxicity. Our results demonstrated that TFS had a significant impact on cell proliferation, as seen by the decrease in MI. The rate of MI could also be decreased through increased apoptosis in meristematic cells. Shi et al.^47^ showed that an activation of caspase-3/caspase-9 in combination with an altered mitochondrial process in the case of strobilurin-type pesticide exposure can lead to an inevitable apoptosis. Alias et al.^15^ suggested that the reduction of root elongation is clearly related to decreased apical meristematic activity and blocked mitosis. In our study, this phenomenon may be a major explanation for suppressed root growth along with other malfunctions in cell metabolism, including oxidative stress. Furthermore, the mitotic cycle disruption may arise from an obstruction in the G1 phase, limited DNA replication in the S phase, spindle failures or alterations in the typical length of the mitotic phases^48^. There are numerous studies showing that root elongation and weight gain are restricted in *A. cepa* due to exposure to pollutants, concomitant with a decrease in MI^49,50^. In a previous study, Macar et al.^6^ demonstrated that higher dosages of TFS caused a dose-dependent decline in MI in *A. cepa* root cells. According to Kang et al.^51^, Gbex inhibited the apoptotic effect of paraquat on PC12 cells by reducing caspase-3 activation. In this context, Gbex may attenuate apoptosis and thus mitigate the mitodepressive effect by suppressing TFS-activated caspase genes.

**Table 3:** Protective efficiency of Gbex against TFS-induced genotoxicity.

Abnormalities	C	Gbex1	Gbex2	TFS	TFS + Gbex1	TFS + Gbex2
DCN MI (%)	860 ± 17.34^a^	874 ± 17.58^a^	867 ± 17.45^a^	620 ± 9.85^d^	687 ± 11.16^c^	735 ± 13.22^b^
	(8.60)	(8.74)	(8.67)	(6.20)	(6.87)	(7.35)
MN	0.00 ± 0.00^d^	0.00 ± 0.00^d^	0.00 ± 0.00^d^	58.7 ± 4.74^a^	50.8 ± 4.35^b^	39.7 ± 3.78^c^
F	0.00 ± 0.00^d^	0.00 ± 0.00^d^	0.00 ± 0.00^d^	53.4 ± 4.51^a^	44.6 ± 3.85^b^	35.7 ± 3.46^c^
V	0.00 ± 0.00^d^	0.00 ± 0.00^d^	0.00 ± 0.00^d^	47.3 ± 4.20^a^	37.5 ± 3.58^b^	29.1 ± 2.81^c^
S	0.16 ± 0.38^d^	0.12 ± 0.28^d^	0.10 ± 0.23^d^	40.8 ± 3.65^a^	32.6 ± 2.96^b^	25.9 ± 2.64^c^
UCD	0.10 ± 0.23^d^	0.14 ± 0.33^d^	0.13 ± 0.30^d^	32.5 ± 2.94^a^	25.4 ± 2.60^b^	18.6 ± 2.15^c^
B	0.00 ± 0.00^d^	0.00 ± 0.00^d^	0.00 ± 0.00^d^	25.2 ± 2.54^a^	17.3 ± 1.98^b^	11.5 ± 1.74^c^
VCN	0.00 ± 0.00^d^	0.00 ± 0.00^d^	0.00 ± 0.00^d^	17.8 ± 1.90^a^	12.2 ± 1.75^b^	7.7 ± 1.21^c^
RP	0.00 ± 0.00^d^	0.00 ± 0.00^d^	0.00 ± 0.00^d^	13.4 ± 1.70^a^	8.8 ± 1.25^b^	4.1 ± 0.84^c^
IM	0.00 ± 0.00^d^	0.00 ± 0.00^d^	0.00 ± 0.00^d^	9.7 ± 1.32^a^	5.8 ± 0.89^b^	2.5 ± 0.48^c^

C: Control, Gbex1: 200 mg/L Gbex, Gbex2: 400 mg/L Gbex, TFS: 0.8 g/L TFS, TFS + Gbex1: 200 mg/L Gbex + 0.8 g/L TFS, TFS + Gbex2: 400 mg/L Gbex + 0.8 g/L TFS. Values (displayed as mean ± SD) in the same line with a different letter (a–d) are significant at *p* < 0.05. MI: mitotic index, MN: micronucleus, F: fragment, V: vagrant chromosome, S: sticky chromosome, UCD: uneven chromatin distribution, B: bridge, VCN: vacuole-containing nucleus, RP: reverse polarization, IM: irregular mitosis, DCN: dividing cell number.

Although MN production was not seen in the C, Gbex1, or Gbex2 groups, the MN level rose to 58.7 ± 4.74 in TFS-treated bulbs (Table 3, Fig. 3a). Chromosome breaks, chromosomal losses and polyploidy, which the cells are unable to repair, typically result in micronuclei^52^. MN serves as a trustworthy indicator of cytogenetic abnormalities that develop after exposure to genotoxic chemicals^53^. Causes of micronucleation include microtubule malfunctions, chromosomal lagging, acentric chromosome fragments and failure to assemble the nucleus and nuclear envelope at the end of mitosis^54^. The findings of our research supported the previous publications of Macar et al.^6^ and Liu et al.^55^, which reported that TFS caused metabolic damage in addition to genotoxicity. In addition, the genotoxicity of strobilurins like azoxystrobin and pyraclostrobin and their ability to cause MN formation have been highlighted in earlier research^56,57^. *A. cepa* root cells in the TFS group exhibited the highest levels of the following CAs among all groups: fragment (Fig. 3b), vagrant chromosome (Fig. 3c), sticky chromosome (Fig. 3d), uneven chromatin distribution (Fig. 3e), bridge (Fig. 3f), vacuole-containing nucleus (Fig. 3g), reverse polarization (Fig. 3h) and irregular mitosis (Fig. 3i). Chromosomal fragmentation and MN formation may be facilitated by substances that not only break DNA double bonds but also inhibit DNA repair^58^. Vagrant chromosomes are the result of irregularities in the organization and usual operation of the spindle apparatus^59^. Due to a rise in chromatin unseparation-related UCD, the number of vagrant chromosomes increases^60^. Stickiness is a permanent defect that results in cell death and represents chemical toxicity. It may result from subchromatid linkages between chromosomes, DNA breakage or depolarization, improper non-histone protein activity during chromosome assembly, physical adhesion of the protein-rich composition of chromatin, or a combination of these factors^14^. Our findings proved that TFS is clastogenic because chromosomal bridges and breaks reflect both direct and clastogenic actions of chemicals on DNA molecules^61^. According to do Carmo Langiano and Martinez^62^, vacuolization of the nucleus, one of the most common CAs upon TFS treatment, can lead to subsequent degeneration of the nucleus and eventual cell death. The CAs-inducing effect of pesticides in studies on non-target organisms has been extensively addressed before^6,63^.

Researchers prefer the comet test for assessing DNA damage because it is sensitive, adaptable and outstanding; the images it generates are so similar to celestial bodies that peering through a microscope at damaged DNA molecules may be as inspiring as stargazing^64^. The comet assay is frequently used in combination with the Allium test to identify genomic imbalances and genetic material damage brought on by a variety of substances in living organisms^65^. The first three groups, comprising control, Gbex1 and Gbex2, displayed negligible damage as measured by tail DNA%. (Table 4). On the other hand, a dramatic rise in DNA tail (%) was induced by TFS exposure (34.4 ± 3.66). Our results demonstrated that TFS treatment led to DNA strand breakage, which was evident by an extreme increase in tail DNA. In addition, Garanzini and Menone^66^ suggested a notable increment in both oxidative stress and DNA fragmentation in azoxystrobin-treated *Myriophyllum quitense*, as shown by CAT activity and the Comet test, respectively. Furthermore, our research unequivocally showed that CAs and comet-proven DNA damage may both be employed as biomarkers with comparable sensitivity to identify damage caused by TFS.

**Table 4:** Comet profile of TFS-induced DNA damage.

Parameters	C	Gbex1	Gbex2	TFS	TFS + Gbex1	TFS + Gbex2
Head diameter (px)	36.000	44.000	38.000	38.000	28.000	40.000
Baş density	273.985	381.770	308.860	191.953	523.371	265.823
Head DNA (%)	99.9 ± 0.32^a^	99.3 ± 1.06^a^	99.4 ± 0.84^a^	65.6 ± 3.66^d^	75.3 ± 3.16^c^	86.7 ± 3.95^b^
Tail length (px)	1.000	1.000	3.000	33.000	57.000	18.000
Tail density	36	2.809	1.748	100.544	171.365	40.919
Tail DNA (%)	0.10 ± 0.09^e^	0.70 ± 0.16^d^	0.60 ± 0.08^d^	34.4 ± 3.66^a^	24.7 ± 3.16^b^	13.3 ± 3.95^c^
Tail moment	0.000131	0.007304	0.016883	14.059	11.343	2.401

C: Control, Gbex1: 200 mg/L Gbex, Gbex2: 400 mg/L Gbex, TFS: 0.8 g/L TFS, TFS + Gbex1: 200 mg/L Gbex + 0.8 g/L TFS, TFS + Gbex2: 400 mg/L Gbex + 0.8 g/L TFS. Values (displayed as mean ± SD) in the same line with a different letter (a-e) are significant at p < 0.05 (n = 10). DNA damage was examined in 1,000 cells from each group.

In comparison to the TFS group, in the groups where Gbex and TFS were co-applied, a restoration in MN, MI, CAs and tail DNA (%) rates was shown as a result of increasing Gbex dosage. Indeed, tail DNA (%) was recorded as 24.7 ± 3.16 and 13.3 ± 3.95 in the TFS + Gbex1 and TFS + Gbex2 groups, respectively (Table 4). Therefore, the application of the higher dose of Gbex mixed with TFS switched the damage level from medium to weak compared to the TFS group. Our results were in line with previous studies showing that Gbex can normalize or reduce the tail DNA in organisms suffering from genotoxicity^67^. The TFS + Gbex2 group showed the greatest increase in MI (735 ± 13.22), the greatest drop in MN (39.7 ± 3.78) and the greatest decrease in CAs, but even in this group, the values of the control group could not be attained. All chromosomal abnormalities in the TFS + Gbex2 group decreased between 33 and 74% compared to the TFS group. The findings of the study by Marques et al.^68^ supported the suggestion that Gbex participates in DNA repair pathways in addition to preventing DNA damage. In addition, Zheng et al.^69^ showed that Gbex alleviated glyphosate-induced toxicity by upregulating genes associated with the immune system, demonstrating that Gbex can exert direct effects on genes. Gbex demonstrates distinct efficacy against hydrogen peroxide, one of the most prevalent ROS in cells experiencing oxidative stress, by scavenging hydroxyl radicals and inducing an antioxidant response in the cells. Therefore, it offers remarkable protection against DNA damage brought on by oxidative stress^68^. The most prevalent phenolic ingredient in Gbex was rutin (vitamin P) (Table 1). Due to its antioxidant involvement in Fenton reactions, rutin has enormous potential for maintaining genomic integrity^70^. According to Webster et al.^71^, rutin modulates DNA damage and inadequate enzymatic repair caused by pollutants to prevent carcinogenesis. Protocatechuic acid, an antioxidant 10 times more potent than α-tocopherol, has various benefits, including boosting cell activity, eradicating mitochondrial disorders, lowering apoptosis and minimizing free radical generation^72^. Li et al.^73^ reported that protocatechuic acid and p-coumaric acid were among the most prevalent phenolic acids in Gbex, which is consistent with our findings. Anter et al.^74^ demonstrated that protocatechuic acid, the second most prominent phenolic compound in Gbex, had protective capacity against the genotoxic hazards of DNA degrading agents. According to our findings, Gbex was also rich in catechins and quercetin. Catechins have a one-electron reduction potential, which makes them efficient free radical scavengers^75^. Moreover, Zhao et al.^76^ pronounced the ability of catechins to provide protection against DNA damage. Catechins, which are powerful antioxidants, can prevent DNA ruptures or base damage caused by radicals through H-atom or electron transfer by eliminating peroxyl radicals^77^. Ellnain-Wojtaszek et al.^78^ suggested that quercetin had the highest antioxidant activity in Gbex, whereas catechin had a more prolonged scavenging effect. Additionally, depending on their antioxidant activity, bioactive isolates like quercetin have been demonstrated to exhibit an antigenotoxic struggle against H_2_O_2_^75^. The findings of our study corroborated the data of other studies that showed the antigenotoxic effects of Gbex against pollutant stress in living organisms and cell culture^40,68,79^.

The effects of TFS and Gbex treatments on biochemical indicators are displayed in Table 5. The doses of Gbex in the Gbex1 and Gbex2 groups utilized had no discernible impact on the biochemical parameters compared to the control group (*p* > 0.05). Contrarily, the TFS group demonstrated lower chlorophyll *a* and *b* pigment concentrations than those of the control group (Table 4). In the TFS group, chlorophyll *a* and chlorophyll *b* contents decreased by approximately 55% and 70%, respectively, compared to the control group values. In situ assessments of chlorophyll *a* and chlorophyll *b* are crucial indicators for determining how plants respond to shifting environmental conditions^80^. Different forms of chlorophyll extracted from medicinal plants were discovered to exhibit antioxidant power^81^. Similar to our findings, Pérez et al.^63^ reported a decrease in chlorophyll *b* content in response to azoxystrobin, another strobilurin-class fungicide. The results of our study demonstrated that TFS, which affects the electron transport system, had a detrimental effect on chlorophyll *a* and chlorophyll *b* levels.

**Table 5:** Protective role of Gbex against TFS-induced biochemical toxicity.

Groups	MDA (µM/g FW)	Proline (µmol/g FW)	Chlorophyll *a* (mg/g FW)	Chlorophyll *b* (mg/g FW)	SOD (U/mg FW)	CAT (OD_240 nm_ min/g FW)
(C) Control	12.4 ± 1.20^d^	18.7 ± 1.78^d^	17.5 ± 1.68^a^	9.44 ± 1.30^a^	40.2 ± 4.16^d^	1.25 ± 0.64^d^
Gbex1	11.5 ± 1.16^d^	17.9 ± 1.72^d^	17.9 ± 1.66^a^	9.38 ± 1.27^a^	41.3 ± 4.25^d^	1.22 ± 0.61^d^
Gbex2	12.9 ± 1.24^d^	19.3 ± 1.80^d^	18.2 ± 1.72^a^	9.55 ± 1.29^a^	39.8 ± 4.10^d^	1.33 ± 0.66^d^
TFS	33.6 ± 2.42^a^	40.5 ± 3.96^a^	7.95 ± 1.13^d^	2.85 ± 0.77^d^	81.5 ± 6.96^a^	3.44 ± 1.38^a^
TFS + Gbex1	26.4 ± 2.15^b^	34.2 ± 3.10^b^	10.7 ± 1.32^c^	4.00 ± 0.92^c^	72.6 ± 6.22^b^	2.80 ± 1.22^b^
TFS + Gbex2	20.3 ± 1.86^c^	26.4 ± 2.75^c^	13.1 ± 1.44^b^	6.48 ± 1.10^b^	60.6 ± 5.75^c^	2.15 ± 0.98^c^

C: Control, Gbex1: 200 mg/L Gbex, Gbex2: 400 mg/L Gbex, TFS: 0.8 g/L TFS, TFS + Gbex1: 200 mg/L Gbex + 0.8 g/L TFS, TFS + Gbex2: 400 mg/L Gbex + 0.8 g/L TFS. Values (displayed as mean ± SD) in the same column with a different letter (a–e) are significant at *p* < 0.05 (n = 10). MDA: malondialdehyde, SOD: superoxide dismutase, CAT: catalase.

Although Gbex application by itself did not result in a statistically significant difference in proline level, an overbearing rise in proline value was found in the TFS group (40.5 ± 3.96) (Table 5). The accumulation of proline, an osmoprotectant, under stress conditions is generally associated with stress tolerance. It actually functions as a component of the antioxidant defense system in plants struggling with oxidative stress as a non-enzymatic antioxidant^82^. Since the accumulation of proline in plant tissues is a reaction to protect the plant from stress-induced damage, it is a clear sign that the TFS group experienced significant oxidative stress. In order to investigate the oxidative stress caused by TFS and the effect of Gbex on this stress, SOD and CAT enzymes, which are important parts of the enzymatic antioxidant defense system, were investigated. MDA, SOD and CAT play an essential role as remarkable biomarkers in researching oxidative stress due to their stability, simplicity of detection and quick reaction^83^. The superoxide anion radical, converted to hydrogen peroxide by SOD, is rapidly decomposed by CAT. The SOD and CAT enzyme activity in the Gbex1 and Gbex2 groups were unaffected by Gbex application statistically (Table 5). However, SOD activity reached 2.03 times and CAT values reached 2.75 times in the 0.8 g/L TFS group compared to values in the control group. These enzymes are crucial components of the enzymatic antioxidant systems established to repair and prevent ROS-caused damage in plants^84^. Kovačević et al.^85^ reported that changes in SOD and CAT activity in *Enchytraeus albidus* are signs of TFS-induced oxidative stress. Similar to our results, Macar et al.^6^ mentioned that SOD and CAT activities were elevated in *A. cepa* upon TFS application. Therefore, TFS prompts the enzymatic antioxidant defense as an inducer of oxidative stress in *A. cepa*. MDA is used as a biomarker of oxidative stress in plants because it is formed from the degradation of polyunsaturated fatty acid parts in lipid membranes due to oxidative damage^86^. In our study, when compared to the control group, Gbex applied alone did not cause any change in the MDA level, while the MDA level in the TFS group increased to 2.71 times (Table 5). Pandey and Rathore^43^ claimed that strobilurins can generate excessive amounts of oxygen radicals that increase MDA levels and cause oxidative stress that damages DNA or cell membranes. Our findings are compatible with previous studies reporting TFS-induced MDA accumulation in non-target organisms^5,6^. Considering all the biochemical data in this study, it can be interpreted that the application of 0.8 g/L TFS to *A. cepa* causes oxidative stress. It is commonly accepted that oxidative stress caused by pesticides can result in genotoxicity. ROS interact with genetic material and cause genotoxicity, leading to various mutations^87^. From this perspective, our results showed that ROS generation and oxidative stress are among the primary causes of TFS-induced genotoxicity.

When increasing dosages of Gbex were applied together with TFS, the negative effects of TFS on all examined biochemical parameters gradually lessened in the TFS + Gbex1 and TFS + Gbex2 groups. According to our findings, Gbex can directly neutralize ROS or activate the antioxidant defense system, which lessens the impact of oxidative stress on *A. cepa*. Enhancement of frontline antioxidant defense or reduction of workloads of already active enzymatic (SOD and CAT) and non-enzymatic (proline) antioxidants may underlie the oxidative stress quenching achieved through Gbex. According to Marques et al.^68^, the bioactive molecules found in Gbex may successfully recycle ROS-scavenging cellular proteins such as CAT and SOD and directly scavenge ROS, in addition to promoting pentose phosphate pathway activity in producing NADPH. Similarly, de Souza et al.^88^ also pointed out that Gbex regulates the expression of antioxidant enzymes that reduce ROS and reactive nitrogen species and thus reduce lipid peroxidation, thanks to its antioxidant properties. In addition, Yalçın et al.^89^ revealed that Gbex treatment suppressed external H_2_O_2_-induced oxidative damage in mice relative to decreased MDA levels.

**Fig. 4:**
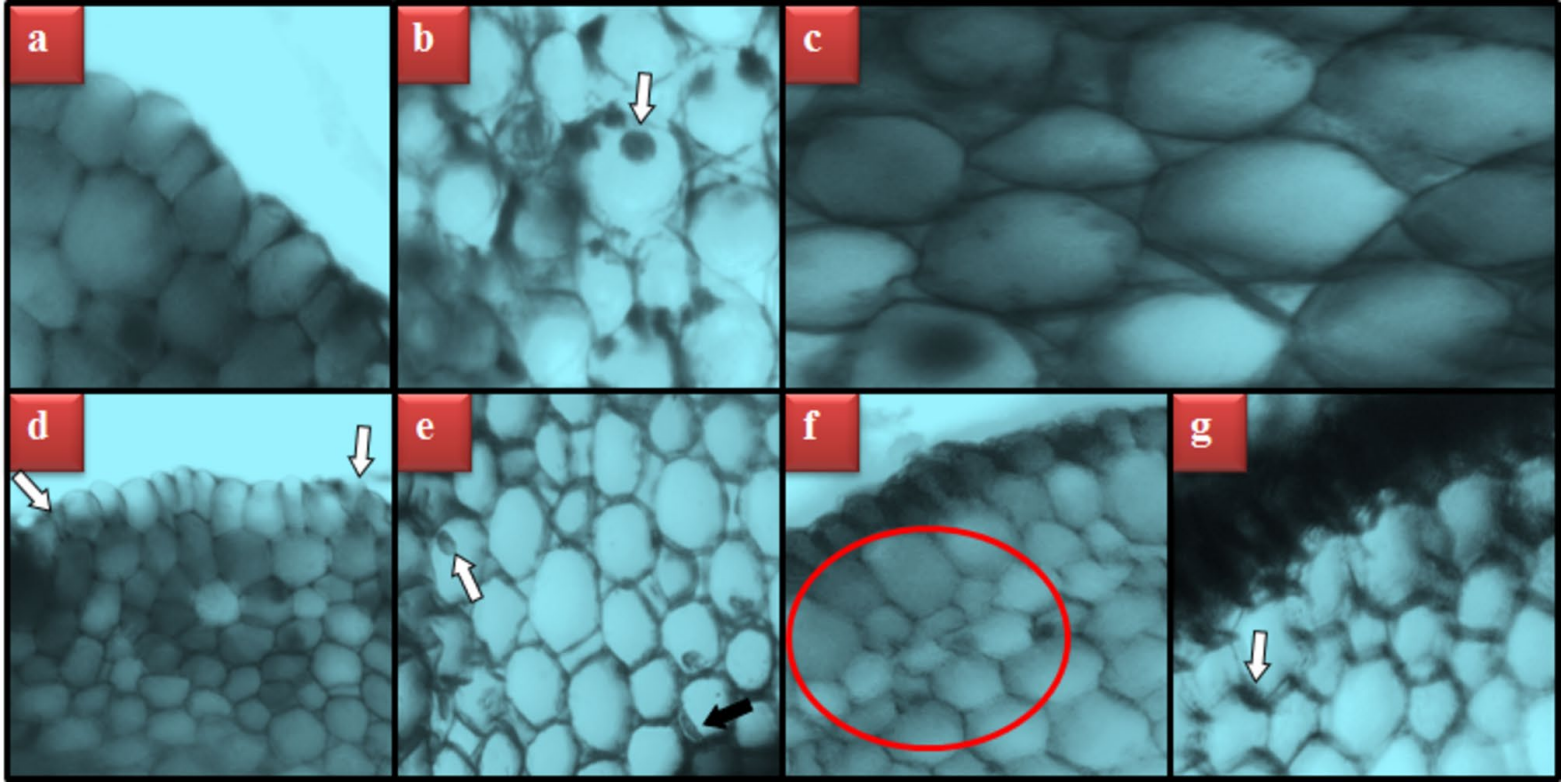
TFS-induced meristematic cell damages. Typical appearance of epidermis cells (**a**), typical appearance of cell nucleus-*oval* (**b**), typical appearance of cortex cells (**c**), epidermis cell damage (**d**), flattened cell nucleus (**e**), cortex cell damage (**f**), thickening of the cortex cell wall (**g**).

TFS administration promoted a series of meristematic cell disorders in *A. cepa* roots (Table 6 and Fig. 4). Gbex treatment did not cause any meristematic cell disorders in the Gbex1 and Gbex2 groups. On the other hand, the 0.8 g/L TFS-exposed group showed a substantial level of epidermis cell damage, flattened cell nucleus, cortex cell damage and thickening cortex cell walls. In the TFS + Gbex1 and TFS + Gbex2 groups, all meristematic cell disorders were gradually alleviated with increasing dosages of Gbex. In the TFS + Gbex2 group, 400 mg/L Gbex treatment reduced epidermis cell damage and flattened cell nucleus to a slight level, while cortex cell damage and thickened cortex cell walls were completely prevented. Previous researchers addressed the structural harm caused by various fungicides to root cells, which are the first parts of the plants exposed to the dangerous compounds^90^. Indeed, Wang et al.^91^ showed that TFS applied in hydroponic culture was found in the roots of *Cucumis sativa* rather than in the leaves and accumulated in the membranes and organelles of root cells. Macar et al.^6^ attributed similar destructive effects of high doses of TFS on the *A. cepa* root meristem to oxidative stress-induced membrane damages and genotoxicity. However, it is unclear if the thickening of the cortex cell walls is a sign of injury or a defensive mechanism to prevent the TFS from reaching higher areas of the plant. As revealed by the genotoxicity and MDA results in our study, Gbex prevents structural damage to the root meristem with its extraordinary antioxidant and antigenotoxic abilities.

**Table 6:** Protective property of Gbex against TFS-induced meristematic cell damage.

Groups	ECD	FCN	CCD	TCCW
(C) Control	**−**	**−**	**−**	**−**
Gbex1	**−**	**−**	**−**	**−**
Gbex2	**−**	**−**	**−**	**−**
TFS	**+++**	**+++**	**++**	**++**
TFS + Gbex1	**++**	**++**	**+**	**+**
TFS + Gbex2	**+**	**+**	**−**	**−**

C: Control, Gbex1: 200 mg/L Gbex, Gbex2: 400 mg/L Gbex, TFS: 0.8 g/L TFS, TFS + Gbex1: 200 mg/L Gbex + 0.8 g/L TFS, TFS + Gbex2: 400 mg/L Gbex + 0.8 g/L TFS. ECD: epidermis cell damage, FCN: flattened cell nucleus, CCD: cortex cell damage, TCCW: thickening of the cortex cell wall. Damage frequency for 100 images expressed as: (−): 0–10 damage, (+): 10–25 damage, (++): 25–50 damage, (+++): 50–75 damage, (++++): 75–100 damage.

## Conclusion

The need for protective functional foods that may be utilized in daily life to defend against pesticides is rising quickly as their use escalates. The present study demonstrated that Gbex exerts a protective effect against phytotoxicity and genotoxicity induced by TFS in *A. cepa*. According to the results of our study, probably due to the antioxidant properties provided by its phenolic content, Gbex increased the survival rate of cells, alleviated oxidative stress, minimized DNA damage, and mitigated the toxic effects of TFS. Gbex has shown extraordinary potential as a functional food that can protect against dangerous agrochemicals. With this study, it has been proven once again that the comet assay, along with micronucleus and chromosomal abnormality assays, are sensitive methods for determining genotoxicity. According to our findings, these tests are also quite successful in investigating the antioxidant and antigenotoxic capacities of potent functional foods.

## Data Availability

All data generated or analyzed during this study are included in this published article.
